# Video-Based Automated Lameness Detection for Dairy Cows

**DOI:** 10.3390/s25185771

**Published:** 2025-09-16

**Authors:** Kamil Szyc, Marta Hebda, Kamil Dembiński, Michał Zdunek, Olgierd Unold

**Affiliations:** 1 Department of Computer Engineering, Wrocław University of Science and Technology, 50-370 Wrocław, Poland; olgierd.unold@pwr.edu.pl; 2 Faculty of Geology, Geophysics, and Environmental Protection, AGH University of Krakow, 30-059 Kraków, Poland; 3 MEGAVET Bolków M. Nowicki & Ł.Oktawiec Spółka Cywilna, 59-420 Bolków, Poland; kamildembinski.lelek@gmail.com; 4 7M AMDG Sp. z o.o., 51-361 Wilczyce, Poland

**Keywords:** applied computing in agriculture, video processing, lameness detection, dairy cattle, visual locomotion scoring

## Abstract

Nowadays, the treatment costs associated with lameness rank second among common diseases of cattle. The standard method for detecting lameness is visual observation of the herd by the farmer. However, these methods are time-consuming and labor-intensive and, due to the qualitative nature of the assessment, involve many discrepancies between different human assessors. This study aims to develop fully automated end-to-end methods for the video-based assessment of lameness in dairy cows using data science. For the study, 832 cows with varying degrees of lameness were recorded. The video recordings were then divided into individual frames, where deep learning detected a single cow and its characteristic anatomical points. A custom 7-point locomotion scoring system, inspired by the commonly used 5-level Sprecher (Zinpro) scale, was introduced and evaluated. This scale was used to assess lameness severity based on processed data, which were analyzed using an expert system, machine-learning methods, and a deep-learning approach. Our solution is based on the analysis of the spine curvature, head position, and distance between pairs of legs. The accuracy of detecting binary lameness (healthy vs. lame) through multiple locomotion features approaches expert-level performance, at 0.821 and 0.872, respectively.

## 1. Introduction

Lameness is a serious welfare concern in all types of farms, although its economic and management impact is most evident in large-scale operations, where it contributes to significant losses and increased culling and replacement rates [1]. A recent extensive review [2] reported that the average prevalence of dairy cows’ lameness across various global studies was found to be 22.8%, with reported prevalence values ranging from 5.1% to 45%. Due to its adverse impact, farmers and researchers have shown a keen interest in developing indicators to identify lame animals immediately and assess the severity of the condition.

Lameness is characterized by a set of clinical signs, including pain, mobility problems, reluctance to stand up or transfer weight from one limb to another, and other issues that may affect the cow’s hoof and higher extremities. Clinical lameness may cause breeding problems, such as a longer calving interval and an increased culling risk, up to 8.4 times more [3]. In Ref. [4], the authors compiled an atlas of 27 disease entities affecting the cow’s toe area alone.

Several traits have been developed to evaluate lameness, including head movements, spinal line observation, overlap of the front and back hoof joints, walking speed, limb abduction, adduction, or stride length [5]. Sprecher’s 5-level system (aka Zinpro) [3] is one of the widely recognized and used visual locomotion scoring methods based on a qualitative assessment of the gait and posture of cattle.

However, visual evaluations are subjective and differences between observers can make the process time consuming and challenging [6]. In addition, hiring skilled personnel is difficult and the increasing size of the herds exacerbates the problem. In this context, fully automated and objective lameness-detection systems may become more popular in the coming years [7].

Among the various methods, video-based lameness-detection techniques appear to be the most promising due to the non-invasive nature of the approach and its affordability for on-farm installation. Various studies have investigated the automation of locomotion scoring and lameness detection using video-based systems. Generally, a three-step process is followed to identify lameness from videos. Firstly, computer vision techniques are utilized to recognize the relevant anatomical landmarks. After that, one or more locomotion traits are computed based on the extracted anatomical landmarks. Lastly, a classifier is trained to score lameness using the extracted locomotion traits as features.

The latest advances in deep learning have provided lameness-detection systems with the ability to accurately locate selected anatomical landmarks, segment objects from the background, and locate and mark keypoints [8,9,10].

**Figure 1:**
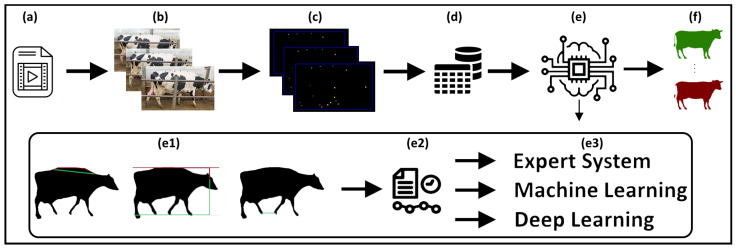
A streamlined pipeline for detecting cow lameness through video analysis. Our solution processes directly from video input to the final lameness-detection decision, embodying an end-to-end system. The process initiates with the capture and input of video into the system (**a**), followed by the separation of the video into individual frames (**b**). Computer vision algorithms are then employed to detect the cow and annotate anatomical landmarks within each frame (**c**). These landmarks and the bounding box data are subsequently stored (**d**). Base algorithms are applied, analyzing the curvature of the spine (**e1—left**), the relative position of the head to the legs and spine (**e1—center**), and the distances between legs on the same side (**e1—right**) for each frame. Finally, the data iare either aggregated or analyzed as a time series (**e2**), utilizing various final decision algorithms (**e3**) to thoroughly evaluate the cow’s health, categorizing it as healthy or lame, or rating it on a 1–7 scale (**f**).

Our study developed an end-to-end pipeline for detecting lameness in dairy cows, utilizing computer vision and machine-learning techniques—see Figure 1. We collected 832 video recordings of cows moving across a camera’s field of view in various Polish cowsheds, ensuring minimal overlap and clear visibility despite challenges such as variable lighting and obstructions. Veterinary experts assigned lameness levels to each cow on a scale of 1–7, using a modified Sprecher scale. Employing the YOLOv5 model for cow detection and the MMPose model for keypoint detection, we analyzed each cow’s movement and body positioning, focusing on critical indicators like the curvature of the spine, the relative position of the head, and the distances between legs on the same side. For each of these indicators, we proposed a “base algorithm”.

We compared three methods for the final detection of lameness: a rule-based expert system, a machine-learning approach (both based on the aggregated results of each base algorithm into a single value per video), and a deep-learning approach (that analyzed the entire sequence of these indicators across frames) which used CNNs and LSTM networks.

Our algorithm evaluates cow lameness on a seven-point scale. To facilitate the evaluation, we also adapted our classifiers for a binary classification system, distinguishing between “healthy” (grades 1–3) and “lame” (grades 6–7) cows. Our veterinary experts agree that this simplified method is suitable for everyday use by cow owners. In comparison, the full seven-grade scale can be used to identify minor lesions in cows immediately.

The lameness-detection algorithm we developed is crucial for several reasons in the context of dairy farming. Although mobility scoring by trained assessors is routinely practiced and often required for welfare assurance, especially on large farms, it remains labor-intensive. Our algorithm helps automate the monitoring of large herds, supporting regular assessments in operations where frequent manual checks can be challenging to sustain. This automation not only streamlines the detection process but also ensures that cows showing early signs of lameness can be identified and treated sooner, significantly improving their welfare and reducing potential productivity losses. Furthermore, the algorithm supports continuous health monitoring, capturing the variability in lameness symptoms over time. Such detailed tracking enables more nuanced interventions and could lead to the development of preventive measures. Lastly, since binary classification is commonly used in existing studies, we also chose to simplify the problem to facilitate comparison with related work.

For end users, the system is intended for routine screening along the passage, producing actionable flags that can be checked during daily herd walks and triage. From an adoption perspective, a single side-view camera and a straightforward setup reduce operational overhead without altering existing handling procedures. This deployment orientation informed model selection and evaluation protocol throughout the paper.

The primary objective of this study is to develop an end-to-end computer vision pipeline capable of reliably detecting lameness in dairy cows under real-world farm conditions. Building on that goal, the main contributions of this work are as follows: (i) The development of a complete pipeline that leverages computer vision and machine-learning techniques to identify lameness in dairy cows. (ii) The identification and analysis of key locomotion-related traits relevant to lameness detection, including spinal curvature, the relative positioning of the head with respect to the legs and spine, and the inter-limb distances on the same side of the body across video frames. (iii) The validation of the lameness-detection results through expert human assessments, providing a reliable ground truth for evaluation. (iv) The investigation of the performance of a machine-learning model for automated lameness detection. (v) The introduction of a novel seven-grade locomotion scoring scale that enables finer resolution for detecting sub-clinical lameness cases, while maintaining backward compatibility with the widely adopted five-point Zinpro.

In terms of novelty, our contribution is primarily methodological integration and clinically grounded operationalization rather than proposing new backbone architectures. Specifically, we (i) define and use an explicit set of interpretable, veterinary-motivated locomotion indicators derived from 22 keypoints (spine curvature, head dynamics, and same-side inter-leg distances), (ii) apply and document a seven-grade, expert-guided ordinal scheme aligned with common farm practice, and (iii) evaluate a single-camera, side-view pipeline across five commercial barns under rigorous metrics (balanced accuracy, Relaxed Accuracy, MSE) with leave-one-out validation.

The paper is organized as follows. Section 2 outlines the overview of our solution. Section 3 reviews existing automated cowshed systems, establishing the context for our approach to detecting lameness. Detailed methodologies and experimental results are presented in Section 4, where we explore the solution’s components, including data management, vision algorithms, base algorithms, and detection algorithms (rule-based expert system, machine learning, and deep learning), alongside experimental outcomes and comparisons with human expert assessments. In Section 5, we discuss the important details and considerations of our system. Section 6 synthesizes our results and underscores our contributions to enhancing automated lameness detection in cows.

## 2. Overall Methodology

We captured video footage of dairy cows moving from one side to the other in front of a camera. The recording strategy and camera system were designed to capture each cow individually, minimizing cow overlaps and ensuring clear visibility for subsequent analysis. These recordings were carried out in various cowsheds in Poland under a range of conditions, including different lighting levels, distances between the camera and the cows, and occasional obstructions such as barriers. Despite these variations, we ensured that each cow traversed a minimum distance of a few meters to ensure consistency in our data collection process. Using a cow-detection model based on the YOLOv5 framework, we identified each instance of a cow crossing in front of the camera, recorded its direction of movement, and discarded any frames not containing a cow. In total, we gathered 832 video recordings of a single cow passing for further analysis in lameness detection.

The recorded videos featuring individual cows were sent to experts for evaluation. These experts assessed the footage, assigning a level of lameness on a scale ranging from 1 (indicating a healthy cow) to 7 (denoting severe lameness), using a modified Sprecher scale as recommended by specialists; see Table 1. Simultaneously, we applied a keypoint-detection algorithm based on the MMPose model to each video to further analyze the cow movements. For every video, we have stored a lameness level, the direction of cow movement, and, for each frame, a bounding box around the cow and 22 keypoints indicating specific parts of the cow’s body.

Both vision models, YOLOv5 and MMPose, were fine-tuned on our dataset, which was manually labeled by our experts. For YOLOv5, the model was trained on a set of 18,491 cow photos and tested on 3218 photos. For MMPose, the final version of the model was trained on 59,106 cow photos and tested on 8420 photos.

Our lameness-detection algorithm, see Figure 1e, performs a comprehensive analysis of each video frame, focusing on key indicators such as the curvature of the spine, the relative position of the head to the legs and spine, and the distances between legs on the same side. Algorithms that calculate the value for a single frame using such an indicator are called “base algorithms”. Veterinary experts were consulted throughout the analysis process to ensure accuracy and relevance.

We employed three distinct methodologies for final lameness detection: a rule-based expert system, a machine-learning (ML) approach, and a deep-learning (DL) approach. The objective of the algorithms was to classify the data on a 7-grade scale for a detailed lameness evaluation or into a binary category of healthy versus lame for a simplified analysis. Table 2 summarizes all algorithms that we used.

For the expert system and the ML approach, we aggregated the data into a single value per base algorithm. The expert system uses rules defined by veterinarians to assess lameness, establishing specific thresholds to evaluate spine, head, and leg distance traits. In the machine-learning model, popular algorithms automatically perform these evaluations based on training data.

In contrast, our DL approach analyzes sequences from the base algorithms calculated per frame without aggregation. This method utilizes Convolutional Neural Networks (CNN) and Bidirectional Long Short-Term Memory (LSTM) networks to evaluate the time series data from spine, head, and leg traits.

We adopted a Leave-One-Out (LOO) evaluation strategy to validate the ML and DL models. This approach ensures the reliability and robustness of our findings by systematically excluding one sample from the dataset during each iteration of the training process, thereby providing a thorough validation mechanism.

Throughout this study, we analyzed 832 videos of cow passages. We tested both scenarios using a 1–7 grade scale (Table 3) and a binary classification (healthy (1–3) vs. lame (6–7) in our scale) (Table 4). For the 1–7 grade scale, the relaxed accuracy (allowing for a one-grade mistake) was 0.736 for the ML model compared to 0.795 for human experts. For binary classification, the accuracy was 0.821 for the ML model and 0.872 for human experts under our LOO protocol.

In summary, YOLOv5 was used to extract bounding boxes for the recorded videos of dairy cows, splitting the videos into segments of individual cow movement crossings. Next, MMPose was employed for keypoint detection in each frame. Then, basic algorithms extracted crucial indicators such as the curvature of the spine, the relative position of the head to the legs and spine, and the distances between legs on the same side. Finally, we tested three algorithms: the Rule-Based and ML approaches, aggregating each indicator’s data from all frames into singular values for the entire cow movement, and the DL approach, which treated the indicators as time series data. These steps provided a comprehensive analysis of expert performance for lameness-detection approaches under our evaluation protocol.

## 3. Related Work

Recently, there has been a surge in the research and development of automated lameness-detection technologies, with the intention of demonstrating the accuracy and practicality of these systems on farms. Comprehensive reviews by Alsaaod et al. [11] have highlighted the different types of available technologies, which can be broadly classified into three or a combination of these categories. Firstly, there are kinetic gait analysis systems that measure the force applied to the cow’s body, using 1-dimensional [12] or 3-dimensional ground reaction force systems [13,14] or pressure-sensitive walkways [15,16,17]. However, kinematic methods are limited by the difficulty of accurately positioning the feet in the measurement units. Secondly, indirect methods that do not involve gait characteristics are used to analyze variables such as thermographic imaging [18], feeding and grooming behavior [19], and social behavior [20]. Lastly, kinematic gait analysis systems that measure changes in the position of specific body segments over time are used. These systems include mainly accelerometers [21] and video-processing techniques [22].

Recently, advances in sensor-based behavior recognition have demonstrated the potential of using inertial measurement units (IMUs) to monitor daily activities of dairy cows, such as lying, standing, walking, feeding, ruminating, and drinking. Ref. [23] developed a machine-learning-based system utilizing IMU data to accurately classify these behaviors, achieving high performance (F1-score 87%) and providing a foundation for early detection of health issues, including lameness.

Vision-processing lameness-detection techniques are proving to be the most promising approach for identifying lameness. These methods are non-invasive, cost-effective, and can be easily installed on-farm. The classification of computer vision systems’ techniques, as per [24], can based on the types of cameras used. These include 2D, 3D, and thermal infrared (TI) cameras. The advanced features of 3D and TI cameras have proven to be more beneficial than traditional 2D cameras. They can capture more information, including image depth and temperature, but in the case of 3D at the cost of greater computational complexity, and in the case of TI combined with other methods [25].

In camera-based lameness detection, the first step is to identify key anatomical landmarks. Background subtraction [8,26], tracking physical markers [27], or markerless pose estimation [28] are used here. In the subsequent stage, an effort is made to identify and distinguish specific traits of animal movement, such as back posture [25,29], step size [30], or head position [31]. Finally, a classifier is trained to assess the degree of lameness utilizing the extracted traits as input features. As in other areas of computer science, there is no established set of machine-learning techniques, and various classification models are being verified; for instance, in Ref. [9] classification model was developed using a random forest algorithm, but the logistic regression, random forest, support vector machines, multi-layer perceptron, and gradient boosting in Ref. [8,10]. Few expert systems can accurately assess the degree of lameness, for example, based on fuzzy logic [32] and probabilistic neural network [33].

A review of the literature suggests that lameness detection is based mainly on a single trait. Only a few studies have used a combination of two traits for detection [8,10,28].

In Ref. [28], researchers developed a fully automated multi-cow lameness-detection system using a deep-learning model called Mask-R-CNN. They used 250 videos of 10 cows and extracted back curvature and head position traits, aggregating them into statistical features. They achieved 98% accuracy on binary lameness detection and 94% accuracy on a 4-point scale lameness scoring with a CatBoost gradient boosting classifier.

The authors of [8] used a combination of traditional and deep-learning-based computer vision to develop a cow lameness-detection system. They employed DeepLabCut to track the location of hoofs and a head in a video of a walking cow and used back curvature and keypoint data to classify the animal as healthy or lame. The Chi-square test revealed that back posture measurement and head bobbing were the most important features and the logistic regression classifier gave the best results with an accuracy of 87.3%. A dataset contained 212 videos and 52 cows.

In Ref. [10], the T-LEAP pose estimation model was used to extract the motion of nine keypoints from 272 videos of 98 unique cows walking outdoors under varying illumination conditions. The trajectories of these keypoints were then used to compute six locomotion traits. Among these traits, the back posture measurement, head bobbing, and tracking distance were found to be the most important. The observers’ scores were merged thoughtfully to improve intra-observer reliability and agreement, which served as the ground truth. Using the synthetic minority oversampling technique (SMOTE), a well-balanced distribution of classes during the training process was guaranteed. By including multiple locomotion traits, classification accuracy improved from 76. 6% with only one trait to 79.9% with the three most important traits and further to 80.1% with the six traits.

In Ref. [34], the proprietary CattleEye system was validated using two-second video snippets (40 frames) captured by overhead cameras positioned above the single-file race that cows traverse as they exit the milking parlour. After segmenting each animal, the software tracks a set of proprietary reference landmarks whose trajectories are passed to an undisclosed convolutional neural network. This network produces a continuous mobility index (0–100), which is subsequently mapped on the four-level AHDB locomotion scale [35]. Weekly system scores were compared with assessments by two experienced veterinarians for 903 cows across three commercial herds, showing good agreement (>80% after collapsing the four-point scale into binary categories 0–1 vs 2–3). A related study [36], involving overlapping authors, evaluated the same system for lameness detection in 7709 cows on 11 UK farms. Although strong agreement with veterinary scores on the AHDB scale was reported, the method remains closed, relies solely on overhead views, and lacks fine-grained locomotion resolution. Both studies also highlight variability between human assessors, an issue further explored in our work. In particular, in both cases, veterinarians evaluated cows live, under the same brief viewing conditions as the algorithm, without access to detailed video or replay. In contrast, our assessments were based on annotated side-view recordings, allowing for more precise and consistent scoring. Our approach analyzes video from 832 cows recorded in five commercial barns, employs a transparent seven-grade clinical scale, and provides methodological disclosure. However, both studies cited demonstrate the effective use of the CattleEye system for lameness detection in commercial farm conditions.

Direct numerical comparisons across studies are limited by differences in datasets, locomotion scales, camera vantage, and evaluation metrics, which collectively shape reported performance figures.

Against this background, our study delineates an explicit, interpretable indicator path and compares three decision paradigms (rule-based expert system, ML, and DL) under the same feature set and side-view setting. This system-level perspective complements prior reports that often emphasize either binary labels or overhead vantage.

Like in Ref. [8,10,28], we propose a lameness-detection method based on more than one trait. We base our evaluation of cow locomotion on a modified Sprecher’s scale into seven levels of lameness and a simplified binary scale (healthy, lame). We used three techniques to evaluate the quality of the classification and verify the evaluation of the veterinarians. The experimental material included images of more than 800 cows, recorded under natural conditions, and was significantly more extensive and more heterogeneous than most of the previously reported in the literature.

## 4. Detailed Methodology and Experimental Results

### 4.1. Data Collection and Evaluation Metrics

We collected data between 2021 and 2023 to analyze cow mobility in five different cowsheds in Poland. Our methodical recording and camera configuration were designed to minimize cow overlap and ensure optimal visibility despite the challenges posed by diverse environmental conditions, including variations in lighting and spatial arrangements. To maintain uniformity and facilitate the whole process, we standardized the videos to a resolution of 960×540 pixels, excluding audio, 30 frames per second, and cut videos to capture individual cows’ passing, thereby guaranteeing both consistency and high quality of the dataset.

We selected a subset of 832 videos to refine our lameness-detection algorithm, representing one of the largest annotated datasets used for this purpose to date. The remaining recordings were used to train our vision models. Each selected video was independently reviewed by veterinary experts, who had access to both the recorded footage and to live observations during data collection. This allowed them to thoroughly assess locomotion, unlike many previous studies that relied solely on brief, real-time scoring. The cows were rated on a scale of 1 to 7 developed specifically for this study (Table 1), with detailed scoring guidelines designed to ensure consistency and capture subtle differences in gait.

We used the scikit-learn library to evaluate cow lameness detection, integrating classification metrics with mean squared error (MSE) to ensure a comprehensive analysis. Our assessment on a 1–7 lameness scale utilized balanced accuracy (Acc), relaxed accuracy (Relaxed Acc), MSE, and recall with precision, adopting a weighted mode. Relaxed accuracy was explicitly designed to permit a +/− 1 grade margin of error, addressing the inherent difficulties in precise lameness grading. This metric is intentionally used to reflect the inter-observer uncertainty documented in our expert study (see Section 5). Furthermore, we applied a binary classification approach to distinguish between “healthy” (grades 1–3) and “lame” (grades 6–7) cows, as recommended by veterinary experts for ease of daily use by cow owners. For this, we used accuracy, recall, and precision in binary mode. A leave-one-out (LOO) cross-validation strategy was used to maximize data utilization.

Table 5 reports pairwise Cohen’s κ across expert pairs and includes one intra-rater comparison (Expert 3a vs. 3b). For orientation, values in the ranges commonly labelled as ‘fair’ to ‘moderate’ agreement are present in our study, which is consistent with the difficulty of seven-grade ordinal scoring. While weighted agreement measures are often used for ordinal labels, in our evaluation the tolerance to near-miss grades is captured explicitly by reporting Relaxed Accuracy (±1 grade) alongside exact-match and MSE.

We use balanced accuracy and a LOO cross-validation protocol to mitigate class imbalance and reduce optimistic bias. Both are stricter than commonly used single-split evaluations and better reflect expected field performance.

Our solution, designed as an end-to-end process from video input to lameness-detection decision, leverages a vision model to extract keypoints and bounding boxes from each frame. These keypoints, defined by their (x, y) positions (in pixels) and frame number, serve as inputs for our algorithms. We utilize “Base algorithms” to transform these inputs into numeric values, quantifying factors such as the curvature of the spine or the distances between legs. Depending on the algorithms, we used numeric values for indicators per frame for DL or aggregated values for the sequence values for ML and Expert System. The summary of algorithms, inputs, outputs, and goals is in Table 2.

### 4.2. Locomotion-Scoring Scheme

We adopted the well-established five-level Sprecher (Zinpro) locomotion score [3] as a starting point, but on the advice of our senior veterinarians, introduced half-point increments to capture the “in-between” cases they routinely observe. Comparable refinements already exist in the literature; [37], for instance, applied a nine-level (0.5-step) scheme that has since been used in multiple experimental studies. Because none of the cows in our five Polish herds showed the extreme impairment corresponding to >4 on the original Sprecher scale, we truncated the sequence to seven grades (1–7, i.e., Sprecher 1–4 at 0.5-point resolution; see Table 1). This trimming (i) removes empty categories, (ii) aligns the labels with the severity range our automated system must recognise in practice, and (iii) retains the extra granularity that improves inter-observer agreement and yields a more precise ground truth for machine-learning models. Finally, to remain comparable with studies that dichotomise the Sprecher scale, we also report a binary mapping of scores 1–3 as healthy and 6–7 as lame following the convention noted by literature [38]. Cows assigned the intermediate locomotion grades 4–5 were omitted from the binary evaluation because, as the paper notes, these borderline cases suffer the lowest inter-observer agreement, which would blur the otherwise clear healthy-versus-lame distinction needed for robust evaluation.

### 4.3. Vision Models

**Figure 2:**
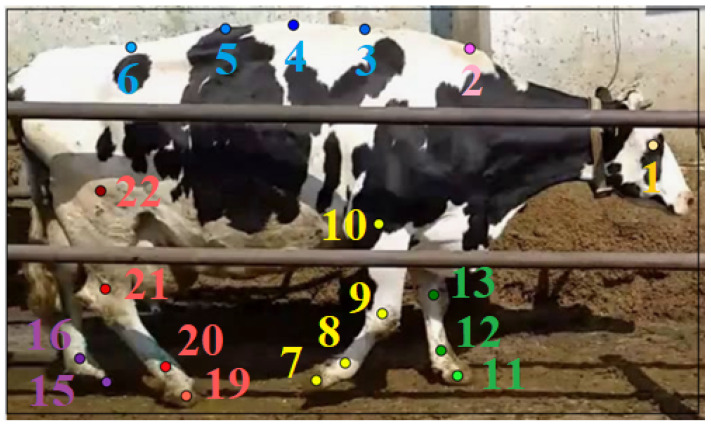
Annotated cow with bounding box and 22 key anatomical points for lameness detection. “FR” denotes Front Right, “FL” Front Left, “BR” Back Right, and “BL” Back Left, indicating specific limbs for precise tagging and analysis. Points are tagged as follows: 1—Eye, 2—Neck, 3—Spine_1, 4—Spine_2, 5—Spine_3, 6—Spine_4, 7—FR_Hoof, 8—FR_Fetlock, 9—FR_Carpal, 10—FR_Elbow, 11—FL_Hoof, 12—FL_Fetlock, 13—FL_Carpal, 14—FL_Elbow, 15—BL_Hoof, 16—BL_Fetlock, 17—BL_Ankle, 18—BL_Knee, 19—BR_Hoof, 20—BR_Fetlock, 21—BR_Ankle, and 22—BR_Knee. Points that were not visible were not tagged—as here 14, 17, 18.

We employed deep-learning vision models for two main tasks: object detection to identify cows and 22 keypoint detection to locate essential animal anatomical markers—see Figure 2 for details. In model selection, we prioritized end-to-end task performance (lameness-detection quality), runtime on edge hardware, and annotation efficiency, rather than maximizing detector/pose mAP alone. For readers less familiar with detection metrics, mean Average Precision (mAP) summarizes how precisely and completely predicted boxes match ground truth across multiple overlap thresholds, integrating precision and recall across operating points (higher is better).

While we employ established detectors and pose estimators, the novelty of this work lies in a coherent integration from detection and keypoints to interpretable locomotion indicators and three decision modules, yielding a transparent signal-to-decision path under farm conditions.

#### 4.3.1. Object Detection

Among the available object-detection solutions, such as the YOLO family, EfficientDet [39], and DETR [40] (based on transformers), we needed a model known for its popularity, scalability, and flexibility in terms of size and speed. Thus, we selected YOLOv5 [41]. Beyond raw accuracy, our detector choice balanced accuracy, inference latency, and model size under edge-deployment constraints relevant to farm settings. In this context, YOLOv5 provided a favorable accuracy-latency trade-off and predictable transfer-learning behavior on our dataset, which facilitated reliable single-cow localization across variable lighting and minor occlusions.

To detect cows in farm video recordings, we used a pre-trained YOLOv5 extra-large model and applied transfer learning by retraining the model on the training set. The model was trained on a set of 18.491 and tested on 3.218 cow photos. To evaluate its effectiveness, we utilized the mean average precision object-detection metric, corresponding to the average AP for IoU from 0.5 to 0.95 with a step size of 0.05 (mAP[0.50:0.05:0.95]). The final result on the test set was 0.938 mAP, which satisfied our predefined detection requirement; consequently, we retained this configuration for all experiments.

#### 4.3.2. Keypoint Detection

For keypoint detection, we used the MMPose library [42], considering state-of-the-art heatmap, top-down options (DeepPose [43], Hourglass [44], HRNet [45], ViPNAS [22]) with FPN [46] and DarkPose [47], as well as common backbones (ResNet [48], MobileNetV2 [49], Swin [50]), alongside alternatives such as OpenPifPaf [51] and DeepLabCut [52]. Ultimately, we opted for a heatmap top-down model combined with HRNet and DarkPose in MMPose, pre-trained on the COCO dataset. We selected this model because it proved robust to partial occlusions, allowed straightforward fine-tuning to our expert-defined keypoints, and integrates cleanly with a detector-tracker pipeline. While alternatives such as OpenPifPaf, DeepLabCut, or keypoint R-CNN/transformer-based methods can be competitive, they typically introduce different engineering trade-offs (e.g., association logic for multi-animal scenes, annotation demands, or memory footprint). Given our single-cow-per-frame acquisition protocol and downstream decision modules, we prioritized consistent keypoint localization at practical runtimes over pursuing marginal gains in pose mAP alone. The model checkpoint based on the COCO dataset was also important because this dataset includes the “cow” category, albeit with points different from those selected by our experts, making it a reasonable basis for subsequent fine-tuning.

The development of the keypoint-detection model within MMPose involved testing different types of models and parameters on a growing dataset. The performance of this problem was assessed using the COCO mAP[0.50:0.05:0.95] metric, which calculates the average precision value for Object Keypoint Similarity from 0.5 to 0.95 in steps of 0.05. The model’s final version was trained on 59.106 cow photos and tested on 8.420 more. The final result for this model was 0.944 COCO mAP. The detailed results for a specific group of points (as shown in Figure 2) were as follows: for spine points (with a neck) was 0.835 COCO mAP, for back left legs was 0.955 COCO mAP, for back right legs was 0.944 COCO mAP, for front left legs was 0.978 COCO mAP, and for front right legs was 0.978 COCO mAP.

### 4.4. Lameness Detection—Base Algorithms

In collaboration with zoological experts, we researched algorithms for detecting lameness. Finally, we selected three algorithms to assess lameness: spine curvature, head position, and hitting the same place with the hooves. The analyses were performed on a single cow passage in the video, with the results of the base algorithms being obtained for each video frame.

#### 4.4.1. Spine Curvature

Veterinary experts have detailed the methodology (as depicted in Table 1) to measure the curvature of the cow spine on a 7-grade scale, where 1 signifies a nearly perfectly straight spine and 7 indicates significant curvature. The algorithm analyzes spine curvature by focusing on the coordinates of spine points within video frames, precisely those captured while the cow is in motion and normalized to the cow’s bounding box. Two approaches were explored to assess the curvature of the spine: the angle and distance ratios.

For the angle ratio, the algorithm fits a circle to the designated spine points and calculates the angle formed by the neck, Spine_2, and Spine_4 points. This angle is normalized by dividing it by 180. The distance ratio approach calculates the ratio between the length spanning the furthest spine points (Neck and Spine_4) and the total length connecting all spine points, as illustrated in Figure 1(e1—left).

**Figure 3:**
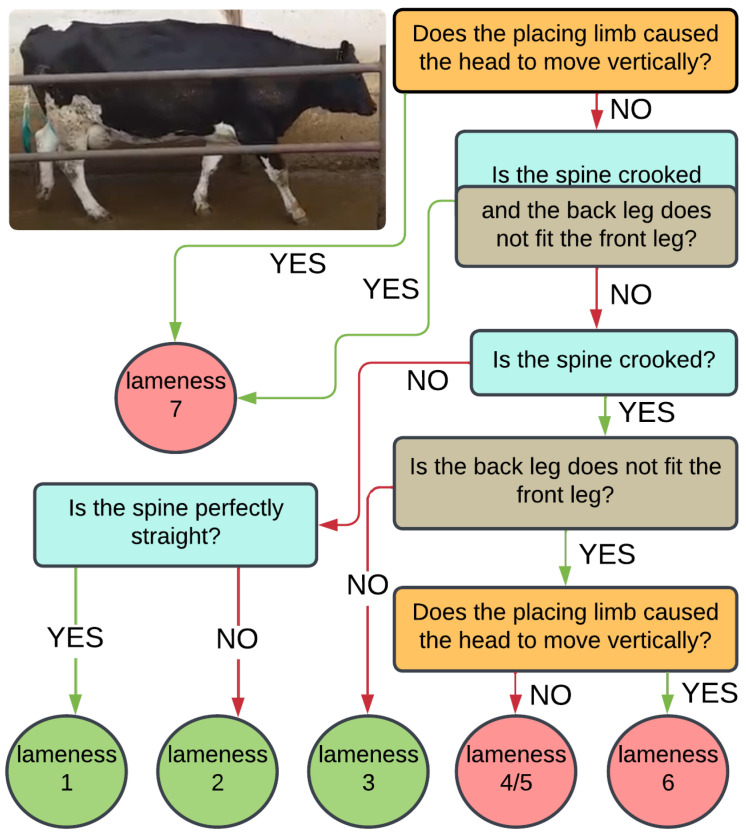
The diagram illustrates the rule-based expert system created by veterinary specialists to determine the degree of lameness. The system employs various algorithms and division thresholds for each division. The lameness grades are determined using three base algorithms: applying the spine algorithm (blue), using the head algorithm (yellow), and hitting the same place with the hooves (grey). The final lameness grades, ranging from 1 to 7, are illustrated in the circular boxes.

For machine-learning algorithms and the expert system, in a further final analysis, we synthesized these measurements into a singular value. This singular is calculated in the following way. We apply temporal smoothing through convolution with a sliding window on the data obtained for each frame, followed by the identification of local minima. The final value for each method is derived from the average of these minima. The algorithm tailors its final result by blending angle and distance in specific ratios for specific splits (see Figure 3).

#### 4.4.2. Head Position

The cow’s head should remain relatively steady during motion for the healthy cow, with little to no vertical movement. For a lameness grade of 6, the cow is expected to show slight vertical head movement upon limb contact with the ground. In cases of a grade 7 lameness score, significant downward head movement is observed when loading the affected limb, which is then lifted along with the head as the limb is relieved. This algorithm normalizes the y-coordinates of head positions by referencing the minimum y-value among hoof points and the maximum y-value among spine points, thus scaling these positions within a normalized range, as shown in Figure 1(e1—center). These values are calculated only upon detection of a step taken by the animal. To aggregate the results into one value from all frames, we applied convolution with a sliding window for smoothing. The algorithm calculates the minimum head position value to differentiate between lameness grades 1–6 and 7. For distinguishing between grades 4–5 and 6, it uses the value from the step’s initial phase minus the minimum value.

#### 4.4.3. Distances Between Legs

This algorithm was designed to check if a cow’s front and back hooves hit the same spot. Zoologists have intuited that lameness can be classified on the basis of this parameter, with a gradual classification—see Table 1. The distances between the back and front legs of the cow were determined taking into account the x coordinates of the Hoof points for the FR-BR and FL-BL pairs of legs while in motion. We used the cow’s height to normalize the x-coordinate values in the video. Distances were measured for a single frame and 10 frames before and after the step. In the data aggregation process, we selected the minimum value for the entire step. Next, the average value of the minimum distances for both pairs of legs FR-BR and FL-BL was calculated for all steps taken during a given passage of the animal. The final result for each video was the average of these two values.

### 4.5. Lameness Detection—Final Decision Algorithms

Our lameness-detection algorithm conducts detailed frame-by-frame video analysis, targeting crucial factors like spine curvature, head positioning, and inter-leg distances. For definitive lameness detection, we implemented three methodologies: a rule-based expert system, machine learning (ML), and deep learning (DL). We include three complementary paradigms to reflect practical needs—an interpretable rule-based expert system, a machine-learning approach that attains the highest agreement under the Relaxed Accuracy metric on the 1–7 scale, and a deep-learning approach that yields balanced precision–recall for binary screening.

Evaluating the expert system, ML, and DL modules on the same set of interpretable indicators within one pipeline provides side-by-side evidence to inform deployment-oriented choices (interpretability versus multi-class agreement versus binary screening).

#### 4.5.1. Rule-Based Expert System

Analysis of the collected results and subsequent consultations with zoology specialists showed that each individual base algorithm could return incorrect results. Our study found that in some cases, individual assessment criteria analyzed separately, such as spine curvature, may not accurately reflect the degree of lameness in animals. Human evaluations were more elaborate in such situations. This problem emphasizes the need for an expert hybrid system that considers the various characteristics of the cow as a whole.

The rule-based expert system diagram is displayed in Figure 3. To classify cow lameness into two ranges at different division levels using different algorithms, we utilized an expert system based on a decision tree. The algorithm divides the degree of lameness based on the animal’s various characteristics. During the first stage, cows are categorized into two groups based on their lameness level: those with a level of 1–6 and those with a level of 7. A head algorithm determines this categorization. If a cow is classified into groups 1–6, the spine and legs algorithm’s curvature is used to verify this value. The lameness is then divided into two groups: 1–2 and 3–6, according to the spine algorithm. The same algorithm is used to classify the cow on a scale of 1 or 2. The division of lameness into stages 3 versus 4–6 is determined by the legs algorithm, while the head algorithm determines the division of 4–5 versus 6. Cows with stages 4–5 of lameness are grouped together due to slight differences between these stages of the disease. The divisions were determined by the thresholds assigned with collaborations with vet experts to each algorithm and the level to divide.

This option is preferable when interpretability and explicit, expert-approved thresholds are required for on-farm decision support.

#### 4.5.2. Machine Learning

Using scikit-learn, we evaluated off-the-shelf classifiers and regressors [53]—Decision Trees [54], AdaBoost [55], XGBoost [56], KNN, and SVR [57]—as representative baselines, selecting the best per metric.

The machine-learning models were fed with the results of three individual base algorithms for each video. To evaluate the models, we used an LOO strategy. Our research tested several machine-learning models, and we selected the most effective.

This option is preferable when optimizing agreement on the 1–7 scale, where it attains the highest Relaxed Accuracy.

#### 4.5.3. Deep Learning

We implemented a Convolutional Neural Network (CNN) with Bidirectional Long Short-Term Memory (BiLSTM) architecture in TensorFlow [58], following the sequence-modeling paradigm in Ref. [59]. The model architecture was built sequentially, starting with 1D convolutional layers that increased in complexity, each with 128 to 320 filters, a kernel size of 3, and a ReLU activation function, designed to extract spatial features from the input. It included bidirectional LSTM layers, with the first having 256 units and returning sequences to capture temporal dependencies, and the second with 512 units to summarize temporal information. The architecture concluded with a dense layer for classification into seven lameness scores. We used a categorical cross-entropy loss and an Adam optimizer. Training and evaluation were carried out using an LOO cross-validation strategy to maximize the exposure of the model to the dataset. This approach allowed for a comprehensive assessment of the model’s performance on unseen data. The input consists of per-frame numeric outputs of the three base algorithms (spine curvature, head dynamics, inter-leg distances). Hyperparameters were optimized with Keras Tuner [60] (learning rate, numbers and sizes of convolutional layers/filters, dropout, LSTM depth), and time-series augmentation with tsaug [61] (noise, smoothing, dropout) was applied.

This option is preferable in binary screening scenarios, where it offers balanced sensitivity and precision.

### 4.6. Evaluation

Our study presents a comprehensive evaluation of lameness detection in cows, focusing on both a detailed 1–7 scale—see results in Table 3—and a binary classification system distinguishing healthy cows (grades 1–3) from lame cows (grades 6–7)—see results in Table 4. All figures are point estimates under LOO validation across data collected from five barns. The analysis spans three distinct approaches and human experts. Note that human experts are denoted by “*” because the figures are the mean of three experts on a 45-video subset used only for the human algorithm comparison. While we report exact-match accuracy on the seven-grade, ordinal scale for completeness, this is a deliberately demanding target: fine-grained grading is affected by inter-observer uncertainty and the ordinal nature of the labels. Therefore, we also emphasize Relaxed Accuracy and MSE as clinically meaningful summaries of agreement on the 1–7 scale. In the screening-oriented binary setting, performance approaches expert-level outcomes under our evaluation protocol. Given the inter-rater variability documented in Table 5, a practical ceiling exists for exact-match accuracy on the seven-grade task. Accordingly, we interpret performance primarily via Relaxed Accuracy and MSE. In the binary screening setting, the best ML and DL models (0.821 and 0.813) are close to the 0.872 achieved by human experts, supporting screening utility despite the seven-grade difficulty.

Beyond raw figures, these results demonstrate the feasibility of a transparent, clinically motivated seven-grade pipeline evaluated across five commercial barns, while approaching expert screening accuracy in the binary use case under our evaluation protocol.

Moreover, we assess success primarily by downstream lameness metrics, not by detector/pose mAP in isolation. Under this criterion, the chosen detection-pose stack proved sufficient for the decision modules, with ML achieving the best Relaxed Accuracy on the 1–7 scale and DL providing balanced precision–recall in the binary setting.

In our lameness detection study, the Expert System (ES) achieved the highest accuracy on the 1–7 scale with a score of 0.408, outperforming the machine-learning (ML) and deep-learning (DL) approaches, which scored 0.237 and 0.381, respectively. However, when considering more nuanced metrics such as Mean Squared Error (MSE) and Relaxed Accuracy, which are crucial for detailed problem assessments, the ML approach proved superior. Specifically, the ML approach demonstrated the lowest MSE at 1.631 and the highest Relaxed Accuracy at 0.736, indicating its overall effectiveness in accurately assessing lameness.

Because supervised learners fit to the provided labels, inter-observer uncertainty in the seven-grade ground truth propagates to training and can inflate variance or depress exact-match performance. This further justifies reporting Relaxed Accuracy and MSE.

For binary classification, the DL approach showed competitive accuracy (0.813) compared to ML (0.821), with DL excelling in recall and precision metrics, making it highly effective for distinguishing between healthy and lame cows. Notably, the best ML and DL models attain accuracies, which are close to the 0.872 achieved by human experts, supporting the utility of the system for routine screening.

Further insights were gained through an ablation study focusing on individual base algorithms within the ML—see Table 6. This study revealed that the spine algorithm was particularly effective across both the 1–7 scale and binary classification scenarios. For instance, the Support Vector Regression (SVR) model of the spine algorithm achieved a Relaxed Accuracy of 0.675 on the 1–7 scale, surpassing the performance of the head algorithm (KNN) and the legs algorithm (AdaBoost). In binary classification, the AdaBoost model of the spine algorithm excelled with an accuracy of 0.795, while the best overall ML performance was observed with the Random Forest model, achieving a Relaxed Accuracy of 0.736. This comprehensive evaluation underscores the potential of integrating multiple algorithms to achieve optimal lameness-detection results.

Our methods were evaluated against the average ratings from three experts who assessed a blind sample of 45 selected films. This task aimed to test the consistency of expert evaluations and compare them with our training data. Despite achieving the highest accuracy (0.452) and relaxed accuracy (0.795) among all methods, the experts’ performance was still below expectations. In the binary classification of healthy versus lame cows, the experts reached an accuracy of 0.872, compared to our best-performing ML XGB algorithm (0.821). These outcomes are on par with those of veterinary specialists, underscoring the intricacies of lameness assessment, which poses a challenge for both automatic methods and humans.

## 5. Discussion

In this section, we cover the key details of our lameness-detection system. We discuss the challenges of establishing ground truth, handling unbalanced data, ensuring system generalizability, integrating the system in production, and addressing privacy considerations.

Ground Truth and Expert Variability

Training and validation labels followed detailed scoring guidelines overseen by senior veterinarians. The average of three experts was used only for the comparative test on a held-out subset of 45 videos, whereas the main supervised learning pipeline relied on consistently curated expert labels.

**Figure 4:**
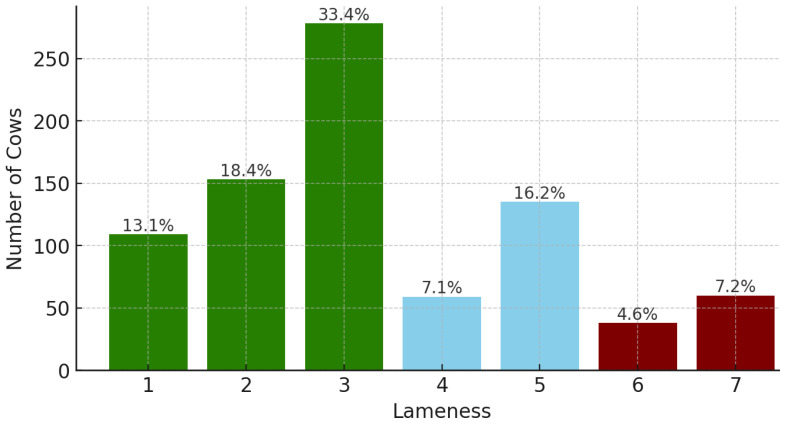
Lameness-detection analysis was performed on a set of 832 cow videos. Within this collection, 638 videos were used explicitly for binary lameness assessment (540 healthy cows and 98 lameness cows).

The problem under consideration is complex due to the uncertainty in ground truth and unbalanced data. The ground truth was established based on individual expert judgments, and the variance in their opinions was significant, as indicated by the Kappa score in Table 5, where a subset of the same 45 cows was evaluated by three experts. The Kappa coefficient measures interrater agreement, with values of 0.75 and above indicating full agreement [62]. Consistent with this variability, we report Relaxed Accuracy as an outcome that explicitly accommodates inter-observer uncertainty. Furthermore, our data set is unbalanced, as shown in Figure 4, highlighting the challenges of managing and interpreting skewed data distributions.

The selected kinematic traits were associated with pain-related gait alterations rather than pregnancy or other conditions. However, to minimize confounding factors, cows in late pregnancy or with clinical conditions were excluded from the dataset.

In our sample, inter-rater agreement spans κ∈[0.23,0.60] across pairs, with an intra-rater comparison of κ=0.60 (Expert 3a vs 3b), indicating moderate internal consistency (Table 6). These figures contextualize the achievable exact-match accuracy on the seven-grade, ordinal scale and motivate reporting tolerance-aware metrics (Relaxed Accuracy) alongside exact-match and MSE.

Given the inconsistency among human experts, defining a gold standard in lameness detection is challenging. To the best of our knowledge, systems similar to ours do not discuss the importance of differences in human evaluations at all. To address this issue, two senior veterinarians prepared detailed guidelines and directly controlled the evaluation process to minimize subjectivity. Most evaluations were conducted or verified by the same senior veterinarian to ensure consistency. In cases of evaluator inconsistency, thorough reviews were conducted to enhance the reliability of our ground truth data.

System Generalizability

Our experiments were conducted in five distinct cowsheds in Poland, showcasing their adaptability to various environments. While our primary focus was on standardized dairy cow production lines, the pose model was pre-trained on COCO and fine-tuned on our labeled barn data, indicating potential applicability beyond the tested scenarios. We acknowledge the limitation of not explicitly considering free-range settings.

The composition of the data set mirrors the real-world prevalence of lameness in dairy cow populations, with a larger representation of healthy cows. We employed balanced accuracy metrics and utilized the Leave-One-Out (LOO) methodology to ensure our algorithms accurately learn characteristics indicative of lameness, addressing potential biases from dataset imbalance.

Direct numerical comparisons with state-of-the-art reports should be interpreted with care, as studies differ in task definition (seven-grade ordinal vs. coarser or binary labels), camera vantage (side vs. overhead), and evaluation protocols. We do not claim one vantage to be universally superior; these design choices entail distinct trade-offs and influence reported headline metrics.

Processing can be performed on the device without continuous network connectivity, which can simplify farm-level operations. We note that increased heterogeneity (five barns, naturally varying conditions) improves external relevance but can depress headline metrics relative to tightly controlled single-site studies, a common trade-off in applied evaluation.

Multiple Cows in Videos

We considered the scenario of multiple cows in a single video. In practice, cowsheds often have gates that naturally separate cows, which mitigates this challenge. Our methodology uses bounding box tracking via YOLOv5 to effectively manage temporary obstructions by other cows. The chosen top-down pipeline mitigates short occlusions and cow intersections, which were a consideration in our model selection. Pilot tests indicated acceptable performance.

While the side-view configuration supports interpretable, locomotion-related features, it is inherently more exposed to intermittent occlusions than overhead layouts under some barn geometries. This trade-off can modestly affect exact-match outcomes without negating screening utility.

Real-Time Processing

Our system was adapted for and tested on Nvidia Jetson, demonstrating its ability to process video data in acceptable time frames and deliver satisfactory results. These deployment constraints informed our model choices: alongside accuracy, we considered latency and memory footprint on embedded devices to ensure practical operation. We therefore emphasized a detector-pose configuration with a predictable accuracy-latency profile for the pipeline. In typical deployments, a single side-view camera positioned alongside the passage suffices, with a basic one-time alignment/calibration during installation. The system produces routine screening outputs that can be reviewed during daily herd checks, which may lower the operational barrier to adoption. We prototyped the pipeline on Nvidia Jetson and observed acceptable throughput in pilot tests. The detailed device-level benchmarks (fps/latency/utilization) are out of scope of this study. We recognize the importance of detailed comparisons and will consider incorporating such analyses in future studies. We have integrated multiple algorithms to improve lameness detection, leveraging each of their strengths.

Privacy Considerations

Our system is designed with privacy considerations in mind, processing only animal recordings without capturing or storing personal data. In production mode, the system does not transmit video footage; instead, it runs vision algorithms locally while extracting and storing only keypoints and bounding box information, minimizing privacy risks. This approach ensures that our system adheres to privacy and security standards, focusing solely on animal welfare assessment.

Ethical Considerations

The animals were observed only in their natural environments without interference in their daily activities. All methods were carried out according to the relevant guidelines and regulations. Our veterinary team and the owners of the animals ensured their well-being throughout the study. No animals were harmed in any way as a result of our observations.

Data Availability

The data that support the findings of this study are available from 7M AMDG, but restrictions apply to the availability of these data, which were used under license for the current study and are therefore not publicly available. Data are, however, available from the authors upon reasonable request and with permission of 7M AMDG. For requests, please contact Michał Zdunek at michalzdunek@gmail.com.

## 6. Conclusions

Drawing a direct comparison between the performance of our lameness classifiers and related works necessitates careful consideration. Although the core task remains the same, the approaches differ significantly between studies, including variations in datasets, locomotion scoring systems, methodologies, and evaluation metrics. It should be mentioned that our study involved one of the largest cow cohorts among similar studies, with each record analyzed from video footage, resulting in a highly detailed and consistent mobility scoring reflecting the conditions of the dairy population. Despite the more complex nature of the data, our approach achieved similar accuracy in detecting binary lameness compared to [10], which also utilized the Sprecher scale, which was merged into two levels, 0.821 vs. 0.801, respectively.

It is important to note that the accuracy of a classifier is directly linked to the precision of its golden standard, and in the case of assessing lameness in cows, a dependable locomotion scale is essential. However, even among experts, there are inaccuracies associated with our scale and the Sprecher (aka Zinpro) scale on which it is based. While the seven-grade, ordinal ground truth introduces inherent uncertainty, it provides clinically meaningful supervision; accordingly, we complement exact-match accuracy with Relaxed Accuracy and MSE and emphasize measures that strengthen labeling consistency (e.g., clear scoring guidelines and expert calibration).

Our research has revealed that the spine is the most critical trait to consider when evaluating lameness in cows, followed by the legs and head. These findings can provide valuable insights for veterinarians, which could lead to more accurate diagnoses and treatments for cows suffering from lameness.

We propose the implementation of an algorithm that can differentiate between healthy and lame cows. To further enhance the performance of deep-learning algorithms, we need to acquire more data. Limitations include label uncertainty on the seven-grade, ordinal scale, class imbalance, and single-view acquisition. Our future work will involve a longitudinal study to monitor changes in cow conditions over time, which our 7-level scale is well-equipped to support.

Taken together, the proposed pipeline supports routine on-farm screening and earlier intervention, which we view as important enablers for adoption in commercial herds. Our novelty is best viewed as methodological integration and clinically grounded operationalization such as a transparent seven-grade, side-view pipeline assessed across five barns, with binary screening performance close to expert level.

## Figures and Tables

**Table 1:** This table presents a refined lameness scoring system based on the Sprecher scale, with adaptations made by veterinary experts to suit the specific needs of our study. The scale starts at 1, equivalent to 1 in the Sprecher scale, and extends to 7, which corresponds to 4 in the Sprecher scale, with each step increasing by 0.5 in Sprecher terms. These descriptions have been developed by veterinary experts to ensure accuracy in identifying various degrees of lameness. It is important to note that scores beyond 7 (4 in Sprecher) were not included, as no cows exhibiting such advanced levels of lameness were encountered in the cowsheds in which we operated. Additionally, we adopted a binary classification approach for some analyses, treating scores 1–3 as indicative of healthy conditions and scores 6–7 as indicative of lameness.

Score	Description
1 (1S)	The cow exhibits a straight spine during movement, with the rear hooves landing in or very close to the front hoof tracks.
2	This score is assigned to uncertain cases where the spine appears nearly straight, indicating minimal lameness. In these instances, the back limbs closely follow the front limbs with minimal deviation.
3 (2S)	The cow’s spine begins to show noticeable curvature during movement, with the back limbs only partially following the front limbs and retracting by a small distance, indicating early signs of discomfort or lameness.
4	A slight curvature of the spine can be observed, with the back limbs not entirely following the front limbs. The back hoof should land on or slightly in front of the heel of the front hoof, indicating mild lameness.
5 (3S)	A curved spine is present, with steps shortened such that the rear hoof lands at or near the heel of the front hoof, indicating moderate lameness. Minimal or uncertain head movement may be observed.
6	The back limbs display a significant deviation from the path of the front limbs, with pronounced stride shortening yet without severe head bobbing, indicating moderate to severe lameness.
7 (4S+)	Clear lameness is observed with visible favoring of a limb, significant head bobbing in the vertical plane linked to limb movement, and unusual limb placement, indicating severe lameness. The back hoof does not follow the front hoof, resulting in an easily noticeable distance.

**Table 2:** Summary of algorithms for detecting lameness in cows.

Algorithm	Input	Output	Description
YoloV5	Video frames	Bounding boxes (bbox)—(x1, y1, x2, y2)	Utilizes deep learning to detect cows within video frames, generating bounding boxes around each cow for further analysis.
MMPose	Video frames	22 keypoints—(x, y) positions	Employ a model to identify specific anatomical keypoints on the cow in each frame, crucial for analyzing cow posture and movement.
Base Algorithms	Sequences of bounding boxes and keypoints for each frame	Numeric values for indicators per frame or aggregated for the sequence	Analyze extracted keypoints and bounding boxes to calculate various indicators like spine curvature, head position, and leg distances. These calculations are critical for assessing lameness.
Expert System	Aggregated numeric values from Base Algorithms	Lameness decision	Uses rules set by veterinary experts to assess lameness based on the aggregated values from the base algorithms, providing a final decision.
Machine Learning	Aggregated numeric values from Base Algorithms	Lameness decision	Apply machine-learning models to the numeric outputs of the base algorithms to classify the severity of lameness.
Deep Learning	Numeric values for each frame from Base Algorithms	Lameness decision	Analyzes time-series data of numeric values from each frame to detect patterns and make a lameness decision using CNNs and LSTM networks.

**Table 3:** A comparison of lameness-detection methods on a scale of 1–7.

Method	Acc	Relaxed Acc	MSE	Recall	Precision
Expert System	0.408	0.704	3.252	0.406	0.479
Machine Learning *	0.237	0.736	1.631	0.303	0.327
Deep Learning	0.381	0.677	2.023	0.381	0.381
Human Experts **	0.452	0.795	1.415	0.496	0.522

* Random Forest; ** The result is based on an average score determined by three experts on the same set of 45 cow videos.

**Table 4:** Comparative analysis of lameness-detection methods for a binary classification of cows: healthy (1–3 in our scale) vs. lame (6–7 in our scale).

Method	Acc	Recall	Precision
Expert System	0.710	0.749	0.881
Machine Learning *	0.821	0.704	0.670
Deep Learning	0.813	0.813	0.813
Human Expert **	0.872	0.844	0.864

* XGB; ** The result is based on an average score determined by three experts on the same set of 45 cow videos.

**Table 5:** The Kappa score results among experts. Each expert scored the lameness of the same 45 cows from the same videos on a scale of 1–7. Expert 3 conducted two separate trials, denoted as ’a’ and ’b,’ to assess consistency within their own evaluations. The results highlight variability in judgment, indicating a lack of consensus even among experts regarding lameness (0.75 and above mean full compliance).

	Exp 1	Exp 2	Exp 3a	Exp 3b
**Exp 1**	-	0.27	0.37	0.37
**Exp 2**	0.27	-	0.31	0.23
**Exp 3a**	0.37	0.31	-	0.60
**Exp 3b**	0.37	0.23	0.60	-

**Table 6:** Evaluation of the impact of individual base algorithms—curvature of the spine, relative head position to legs and spine, and inter-leg distances on the same side—on lameness detection using the machine-learning (ML) approach. The tests were conducted on a full scale and also on a binary scale. Among various ML algorithms tested (AdaBoost, XGB, KNN, SVR), only the top-performing methods are showcased, highlighting their effectiveness in identifying lameness based on singular data sources. To compare data when we used all base algorithms, see the ML row in Table 3 and Table 4.

Scale of 1–7			
**Method**	**Acc**	**Relaxed Acc**	**MSE**
ML Spine SVR	0.256	0.675	1.931
ML Head KNN	0.158	0.526	2.922
ML Legs AdaBoost	0.202	0.519	2.552
**healthy (1–3) vs. lame (6–7) **			
**Method**	**Acc**	**Recall**	**Precision**
ML Spine AdaBoost	0.795	0.653	0.621
ML Head XGB	0.615	0.286	0.483
ML Legs KNN	0.649	0.337	0.611

## Data Availability

Data are available from the authors upon reasonable request and with permission of 7M AMDG. For requests, please contact Michał Zdunek at michalzdunek@gmail.com.
